# Aspirin in combination with gastrodin protects cardiac function and mitigates gastric mucosal injury in response to myocardial ischemia/reperfusion

**DOI:** 10.3389/fphar.2022.995102

**Published:** 2022-09-27

**Authors:** Zhiwu Dong, Lin Yang, Jianlin Jiao, Yongliang Jiang, Hao Li, Gaosheng Yin, Ping Yang, Lin Sun

**Affiliations:** ^1^ Faculty of Basic Medical Science, Kunming Medical University, Kunming, Yunnan, China; ^2^ Department of Cardiology, The Second Affiliated Hospital, Kunming Medical University, Kunming, China; ^3^ Technology Transfer Center, Kunming Medical University, Kunming, China

**Keywords:** myocardial ischemia/reperfusion injury, gastric mucosal injury, Aspirin, Gastrodin, pyroptosis

## Abstract

Myocardial ischemia/reperfusion (I/R) injury after percutaneous coronary intervention (PCI) is common in acute myocardial infarction. Aspirin is commonly prescribed as anti-thrombotic therapy with coronary heart disease (CHD). However, long-term use of aspirin causes severe gastric mucosal damage. Gastrodin is a Chinese natural medicine with anti-inflammatory and anti-oxidative properties. In this study, we investigated the effects of combined therapy with aspirin and gastrodin on the myocardial and gastric mucosal injury in response to myocardial I/R injury and underlying mechanisms using the Sprague-Dawley (SD) rat model. Our results demonstrated that myocardial I/R caused significant cardiac dysfunction and gastric mucosal damage. Administration of aspirin led to significantly reduce myocardial infarction size and myocardial enzyme release, as well as significantly improved cardiac function through exerting anti-inflammatory effects. However, aspirin exacerbated gastric mucosal damage by increasing the levels of inflammatory mediators and endothelin (ET) while reducing prostaglandin E2 (PGE2) levels. The combined treatment with aspirin and gastrodin not only significantly protected gastric mucosa by normalizing the expression levels of the inflammatory factors, ET and PGE2, but also significantly reduced myocardial infarction size and improved cardiac function by inhibiting inflammation in response to I/R. The combination therapy also dramatically down-regulated the levels of pyroptosis-related proteins in the myocardium and gastric mucosa. The combination therapy showed obviously reduced level of thromboxane B2 (TXB2), which was simultaneously accompanied with increased levels of the tissue plasminogen activator (t-PA). This suggested that gastrodin did not inhibit the anti-thrombotic function of aspirin. Accordingly, aspirin in combination with gasrtodin protected the structural and functional integrity of the heart and stomach by suppressing pyroptosis and inflammation. Therefore, combination of aspirin and gastrodin is a promising treatment for cardiac dysfunction and gastric mucosa injury after myocardial I/R.

## Introduction

Acute myocardial infarction (AMI) is leading causes of death worldwide. It is caused by myocardial necrosis resulting from blockage of coronary artery and is associated with arrhythmia, cardiac arrest, and even death. Myocardial reperfusion by percutaneous coronary intervention (PCI) is the common treatment strategy for AMI. However, restoration of the blood flow in the coronary artery by PCI causes myocardial ischemia/reperfusion (I/R) injury (Heusch, 2020). Therefore, antiplatelet drugs such as aspirin are prescribed to prevent thrombosis and secondary myocardial infarctions (Guirguis-Blake et al., 2022).

Aspirin, also termed as acetylsalicylic acid, is the mainstay of anti-thrombotic therapy for coronary heart disease (CHD). It is rapidly absorbed by the gastrointestinal tract following oral administration. The primary pharmacological activity of aspirin includes non-selective inhibition of the cyclooxygenase enzymes (COX), namely, COX-1 and COX-2. Aspirin exerts anti-inflammatory effects by inhibiting the activity of COX-2; while the anti-thrombotic effects of aspirin are exerted by the irreversible inhibition of COX-1 and reduced synthesis of thromboxane A2 (TXA2), which are required for platelet activation. However, long-term use of aspirin causes gastric mucosal injury, gastritis and ulcers (Zareef et al., 2022). Our study confirmed that gastric mucosal injury in response to blood flow restoration after AMI was caused by both aspirin and I/R. The gastric mucosal damage was caused by dysregulation of the protective barrier because of reduced prostaglandin E2 (PGE2) levels, topical injury caused by the acidic properties of aspirin, insufficient blood supply in the gastric mucosa, increased tendency of bleeding and COX-2-induced inflammation (Sostres et al., 2010). To overcome the adverse gastrointestinal effects of aspirin, therapy with enteric-coated aspirin (ECA) and gastro-protective agents like proton pump inhibitors such as rabeprazole and omeprazole are available. However, studies have shown that treatment with ECA does not significantly reduce the risk of gastrointestinal complications (Goldstein et al., 2016; Kedir et al., 2021). Moreover, though combination therapy with aspirin and proton pump inhibitors alleviates gastric mucosal damage, it is associated with adverse effects such as hypomagnesemia and osteoporosis, which thereby affects the treatment of gastric injury (Bridoux et al., 2022). We would have to face the dilemma that how to reduce the side effects of aspirin while taking advantage of its cardiovascular protective effects.

Gastrodin is commonly used to treat cardio-cerebrovascular diseases. Previously, we reported that gastrodin mitigates pyroptosis by inhibiting the nucleotide- binding oligomerization domain, leucine-rich repeat and pyrin domain- containing 3 (NLRP3)/caspase-1 signaling pathway, and further protects myocardium from myocardial I/R injury (Sun et al., 2019). Not only that, it was also demonstrated that gastrodin possesses diverse pharmacological properties including restoration of lysosomal function and autophagy (Tao et al., 2021), inhibition of nuclear factor kappa-B (NF-κB) and mitogen-activated protein kinase (MAPKs), downregulation of inducible nitric oxide synthase (iNOS) and COX-2, and activation of phosphatidylinositol-3-kinase (PI3K)/protein kinase B (Akt) signaling pathway (Yang et al., 2013). In addition, it is reported that gastrodin alleviates betahistine-induced gastrointestinal dysfunction by reducing gastric acid secretion (Zhang et al., 2022b). Meanwhile, it is shown that gastrodin mitigates gastric mucosal damage caused by borneol (Cai et al., 2014). However, how gastrodin takes protective effects on aspirin-induced gastric mucosal injury and the specific mechanism remains unclear. Therefore, we used aspirin in combination with gastrodin as an innovative treatment in this study.

Pyroptosis is defined as an inflammation-related cell death. It is triggered by inflammasomes and is executed by the gasdermin proteins. The classical pyroptosis is mediated by the NLRP3/caspase-1/gasdermin D (GSDMD) pathway (Zhang et al., 2022a). Pyroptosis contributes to myocardial I/R injury, which is characterized by infarction size enlargement and inflammatory cell death mediated by inflammasomes (Toldo et al., 2018). Gastrodin ameliorates microvascular reperfusion injury-induced pyroptosis by inhibiting NLRP3/caspase-1 pathway (Sun et al., 2019). However, the therapeutic effects of gastrodin in combination with aspirin on have not been reported for myocardial and gastric mucosal injury in response to myocardial I/R. Therefore, we investigated if combined treatment with aspirin and gastrodin protected against myocardial and gastric mucosal injury in the myocardial I/R injury and the underlying mechanisms.

## Materials and methods

### Regents

Gastrodin (pure: ≥ 98%) was presented by Kunming Pharmaceutical Group Co.; aspirin was purchased from Ogina Pharmaceutical Co. (Shen Yang, China); rat COX-1 and COX-2 ELISA Kits, rat CK-MB and cTnT ELISA Kits, rat TNF-α and IL-6 ELISA Kits were provided by Cusabio Biotech Co. (Wu Han, China); BCA Protein Assay Kit, Hematoxylin & Eosin (H&E) Staining Kit and TTC solution were provided by Beyotime Biotechnology Co. (Shang Hai, China; Cat. No.: P0010, C0105M); O.C.T. Tissue-Tek was provided by Kang cheng Biological Co. (Shang Hai, China; Cat. No.: 4583); COX-1, COX-2, NLRP3, ASC, caspase-1 and GSDMD-N monoclonal Antibodies were obtained from Affinity Biosciences Co. (Jiang Su, China; Cat. No.: AF7002 and AF7003); GAPDH and β-actin monoclonal Antibodies were obtained from Santa Cruz Co. (Santa Cruz, America; Cat. No.: sc-47724); Fluorescent monoclonal Antibodies from Mouse were purchased from Proteintech Group Co. (Wu Han, China; Cat. No.: CL488-66061); DAPI and polyvinylidene fluoride membrane were obtained from Beyotime Biotechnology Co. (Shang Hai, China). All other experimental supplies were purchased from Sigma.

### Animals

Male Sprague Dawley (SD rats) which were provided by Kunming Medical University were used [Animal Permit No. SCXK (DIAN) K2020-0004]. Inclusion criteria were: The male SD rats were 11 weeks old and the body weight of each rat was between 250 and 300 g. The animals were kept at controlled temperatures (22 ± 1°C) and humidity (45%–55%) on a 12 h light-dark cycle and in an environment free of specific pathogens. The SD rats in this study were provided with normal diet and water. All animal experiment procedures were conducted in accordance with the guidelines of the Animal Ethics and Experimentation Committee of Kunming Medical University and were compliant with to the “Guide for the Care and Use of Laboratory Animals” (revised 1996).

### Drugs administration

In this study, drug preconditioning were used to investigate the effect and mechanism of aspirin and gastrodin in myocardial I/R injury. According to equivalent dose conversion between humans and rats, 50 mg/kg aspirin was used for the study (Huang et al., 2004; Calvieri et al., 2022). 100 SD rats were randomly divided into two parts. One of which was used for the experiments of gastrodin treating gastric mucosal injury induced by aspirin, using 50, 200 and 400 mg/kg doses of gastrodin, were performed to measure the optimal dose of gastrodin used in subsequent experiments. 75 SD rats were randomly divided into Sham group (saline, once a day for 1–5 days), 50 mg/kg aspirin group, aspirin + gastrodin (50, 200 and 400 mg/kg) group, with three mice each. Aspirin was administrated by intragastric injection, and gastrodin was administrated by intraperitoneal injection, once a day for 1–5 days. Another one of which was used for exploring the protective effect of aspirin combined with gastrodin in myocardial I/R. 25 SD rats were randomly divided into Sham group (saline, once a day for 3 days), I/R group (saline, once a day for 3 days; ischemia 45 min/reperfusion 2 h), I/R + 50 mg/kg aspirin group, I/R + aspirin + 200 mg/kg gastrodin group and I/R + 200 mg/kg gastrodin group, with five mice each. Aspirin was administrated by intragastric injection and gastrodin was administrated by intraperitoneal injection, once a day for 3 days. Aspirin and gastrodin were dissolved in 0.9% sodium chloride and stored at 4°C. The SD rats of all the experimental groups were administered the drugs or saline at the same time every day before establishing myocardial I/R model. All procedures were performed in agreement with the U.S. National Institutes of Health Guide for the Care and Use of Laboratory Animals and the China laboratory animal-Guideline for ethical review of animal welfare. All efforts were made to minimize mice suffering and reduce the number of mice used.

### Myocardial ischemia/reperfusion model in rats

Myocardial ischemia was caused in the rats by ligating the left anterior descending coronary artery. Briefly, SD rats were intraperitoneally injected with 3% sodium pentobarbital (3 ml/kg) for anesthesia. Tracheal intubation was performed in a supine position through the mouth. A small animal ventilator was connected externally during the procedure. Myocardial ischemia was detected by electrographic changes using the ECG II leads. The chest skin was disinfected and cut through the fourth intercostal space along the left margin of the sternum. The chest cavity was opened and the exposed pericardium was dissected. The left anterior descending coronary artery was ligated with a 6–0 suture for 45 min. After reperfusion 2 h, blood was collected from the left ventricle and heart was harvested. Subsequently, a 2 cm incision was made in the left upper abdomen and the stomach was harvested. The heart and stomach tissues of all the rats were rinsed in phosphate buffered saline (PBS), fixed in 4% paraformaldehyde, and stored at −80°C. After 4 h at room temperature, the whole blood from the left ventricle was centrifuged at 800 g (3000 rpm) for 30 min at 4°C. The serum samples were extracted and frozen at −80°C for further use. In the Sham group rats, thoracic cavity was opened to expose the left anterior descending branch of the coronary artery and the surgical line was passed through the sub-coronary vessels without ligation.

### Gastric mucosal injury score

Byron gastric mucosal injury scores were evaluated as previously described (Cryer and Feldman, 1999) and are shown in Table 1.

**TABLE 1:** Byron gastric mucosal injury score.

Score(s)	Degree of injury
0	Normal or erythema
1	Any amount of submucosal hemorrhage or edema without erosions
2	1 erosion ± submucosal hemorrhage or edema
3	2–4 erosions ± submucosal hemorrhage or edema
4	5 or more erosions and/or a single ulcer ± submucosal hemorrhage or edema
5	Multiple ulcers ± submucosal hemorrhage or edema

### Hematoxylin and eosin staining

The gastric tissue was fixed in 4% paraformaldehyde for 48 h followed by dehydration in formaldehyde solutions with 15% and 30% sucrose for 48 h. Then, the gastric tissues were incubated twice in the OCT embedding agent for 24 and 4 h, respectively. The embedded tissues were fixed, cut into 5–7 μm thick gastric tissue sections with a frozen slicer, and preserved at −20°C before use. The gastric tissue slices were placed at room temperature for at least 30 min, washed thrice with PBS, stained using the Hematoxylin-Eosin staining Kit according to the manufacturer’s instructions, rehydrated with serial alcohol solutions, clarified with xylene and sealed with neutral balsam. The slices were then observed and imaged using digital light microscopy.

### TTC staining

SD rats were anesthetized with 3% sodium pentobarbital (3 ml/kg). The heart was harvested and rinsed with saline. The left ventricle was cut into 2 mm thick slices, stained in freshly prepared 2% TTC phosphate buffer at 37°C in the dark for 30 min and fixed in 4% paraformaldehyde solution. Image J software was used to analyze the infarct areas. The infarct areas were white and the non-infarct areas stained dark red.

### Enzyme linked immunosorbent assay

The blood samples were stored for 4 h at room temperature and centrifuged at 800 g (3000 rpm) for 30 min at 4°C. Then, the serum was separated and used for the Enzyme linked immunosorbent assay (ELISA) assay. The serum samples were incubated with the antibodies in the wells for 30 min at 37°C. Then, the samples were washed with the wash buffer concentrate for 30 s for 5 times. After removing the buffer, chromogen solution was added to each well and the plates were incubated for 10 min at 37°C. The reaction was terminated by adding the stop solution. Finally, the optical density (OD) was measured in a Thermo Scientific Microplate Reader at 450 nm within 15 min.

### Evaluation of cardiac function

After the modeling, thoracic cavity of the male SD rat was rapidly opened to fully expose the aortic arch. The whole heart was removed along the lower edge of the aortic arch and placed in a pre-chilled K-H solution (0–4°C) to stop the heart beating. At the same time, the heart was gently pressed to expel all the blood from the heart cavity. Then, the Langendorff instrument was set up with the perfusion pressure and intraventricular pressure pressure at zero. After opening the left atrial appendage, the balloon was inserted into the left ventricle. Then, the Langendorff system parameters were set for a constant temperature of 37.5°C, constant pressure of 60–70 mmHg and left ventricular pressure of 10 mmHg. A gas mixture of 95% O_2_ and 5% CO_2_ was continuously injected into the perfusion fluid. Perfusion was continued for 45 min after ensuring stable heartbeat and uniform heart rhythm. Finally, the cardiac function data was analyzed.

### Western blot analysis

The total gastric and heart tissue protein extracts were prepared using the RIPA lysis buffer. Total protein was quantified using the BCA assay kit. Equal amounts of total protein lysates were separated by SDS-PAGE gel electrophoresis. The separated proteins were transferred onto the PVDF membranes. The blots were blocked for 2 h. Then, the blots were incubated overnight at 4°C with the following primary antibodies: anti-COX-1 (dilution: 1:2000), anti-COX-2 (1:2000), anti-NLRP3 (1:2000), anti-ASC (1:2000), anti-caspase-1 (1:2000), anti-GSDMD (1:2000), and anti-GAPDH (1:6000). The blots were washed 3 times with TBS-T buffer for 25 min and incubated with a horseradish peroxidase (HRP)-conjugated antibody for 1.5-2 h at room temperature. The blots were developed with the ECL reagent and the Chemiluminescence imaging system. The protein bands were quantified using the Image J software.

### Immunofluorescence microscopy

The frozen gastric tissue slices were placed at room temperature for 30 min. Then, they were rinsed thrice with PBS for 5 min each. The slices were then incubated with methanol plus hydrogen peroxide solution (9:1 ratio) for 20 min to block the endogenous peroxidase activity. The tissue slices were permeabilized with 0.5% Triton X-100 for 10 min, blocked with 5% goat serum for 2 h at room temperature, and incubated overnight at 4°C with primary antibodies against COX-1 (1:500) and COX-2 (1:500). The following day, the tissue slices were washed with PBS and incubated with fluorescent-conjugated secondary antibodies for 2 h. Finally, the slices were stained with DAPI for 5 min at room temperature and imaged using the fluorescence microscope.

### Statistical analyses

All statistical analyses were performed using GraphPad Prism 8.0.1 software (Inc., La Jolla, San Diego, CA, United States). The data are expressed as the mean ± standard deviation (SD) from at least three replicates. One-way ANOVA followed by post hoc comparisons (Students-Newman-Keuls) were performed for comparing the differences among the groups. All comparisons with a *p < 0.05* were considered statistically significant.

## Results

### Aspirin alleviates myocardial ischemia/reperfusion-induced cardiac dysfunction by suppressing inflammation

**FIGURE 1:**
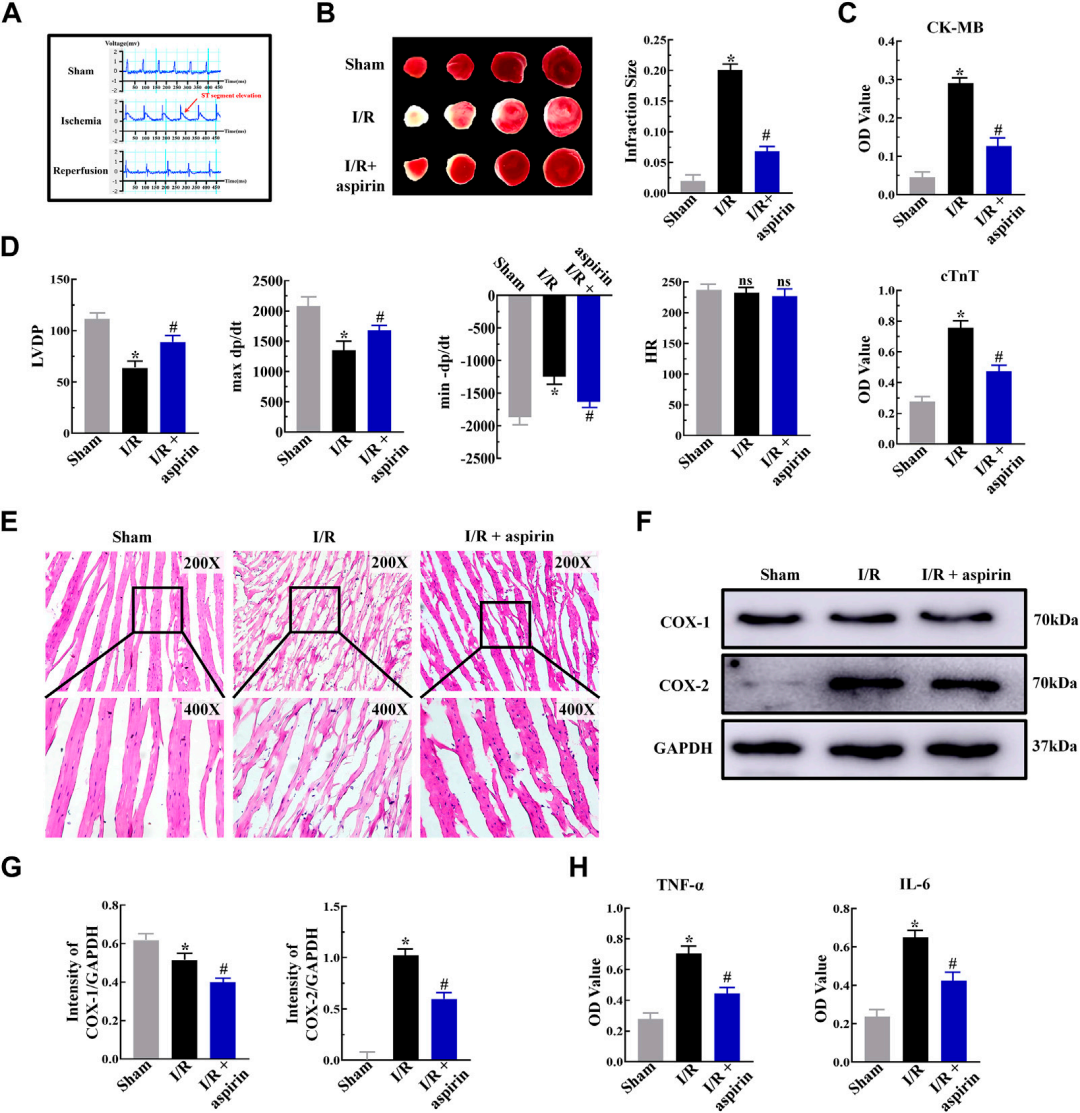
Aspirin inhibits myocardial inflammation and alleviates cardiac dysfunction in response to I/R injury. **(A)** Representative electrocardiogram shows the successful establishment of myocardial I/R injury model (*n* = 5 per group). The red arrows represent changes in the ST segment. **(B)** Representative images show TTC staining of the myocardium from rats (*n* = 5 per group) belonging to the sham, I/R, and I/R + 50 mg/kg aspirin groups. The infarct size quantification of the 3 groups of rats is also shown. **(C)** ELISA assay results show the serum levels of creatine kinase-MB (CK-MB) and cardiac troponin T (cTnT) in the sham, I/R, and I/R + aspirin group rats (*n* = 5 per group). **(D)** Laggendorff *in vitro* cardiac perfusion results show the left ventricular developed pressure (LVDP), maximum and minimum rates of pressure development (max dP/dt and min dP/dt), and heart rate (HR) in the sham, I/R, and I/R + aspirin group rats (*n* = 5 per group). **(E)** Representative H&E staining images (×200 and ×400) show the histological details of the myocardium from the sham, I/R, and I/R + aspirin group rats (*n* = 5 per group). **(F,G)** Representative immunoblots and quantification of the expression levels of COX-1 and COX-2 in the sham, I/R, and I/R + aspirin group rats (*n* = 5 per group). **(H)** ELISA results show the tumor necrosis factor-α (TNF-α) and interleukin-6 (IL-6) level in the myocardial tissues of the sham, I/R, and I/R + aspirin group rats (*n* = 5 per group). The tissues were harvested 2 h post-reperfusion. The data are represented as means ± SD; *p < 0.05 vs. the sham group; ^**#**^ *p < 0.05* vs. the I/R group.

Aspirin is an effective anti-thrombotic drug for cardiovascular diseases. However, the efficacy of aspirin as a canonical non-steroid anti-inflammatory drug to treat myocardial I/R injury is not well established. We pretreated SD rats with 50 mg/kg aspirin for 3 days before building myocardial I/R injury (ischemia 45 min/reperfusion 2 h). The modeling of I/R injury was deemed successful when the ST segment elevation was not normal and showed an “arched back” phenotype (Figure 1A). TTC staining demonstrated large infarct areas in myocardial tissue of the untreated model rats, while pretreatment with aspirin significantly decreased the myocardial infract size and reduced the serum levels of myocardial enzymes such as creatine kinase-MB (CK-MB) and cardiac troponin T (cTnT) after myocardial I/R injury (*p < 0.05*) (Figures 1B,C). We further investigated the effects of aspirin on cardiac function. The left ventricular developed pressure (LVDP), the maximum rise of the left ventricle (+dP/dt) and maximum drop of the left ventricle (−dP/dt) were restored by aspirin (*p < 0.05*), while heart rate (HR) changes were no statistical difference (*p > 0.05*) (Figure 1D **)**. This suggested that aspirin alleviated cardiac dysfunction in I/R injury. Next, we investigated the mechanism by which aspirin protected against cardiac dysfunction. H&E staining showed extensive damage in myocardial tissue of the untreated I/R injury rats, including myocardial structural changes, high infiltration of inflammatory cells and interstitial edema, whilst administration of aspirin mitigated rupture of the myocardial fibers and decreased the infiltration of inflammatory cells (Figure 1E). Aspirin is known to non-selectively inhibit COX enzymes, COX-1 and COX-2, which regulates inflammation. Therefore, we analyzed the expression levels of COX-1, COX-2, interleukin (IL-6), and tumor necrosis factor α (TNF-α) in myocardial tissues. Aspirin significantly reduced COX-1, COX-2, TNF-α and IL-6 expression levels in the cardiac tissues of I/R injury rats compared to the corresponding controls (*p < 0.05*) (Figures 1F–H). This suggested that aspirin protected against cardiac dysfunction by suppressing inflammation during myocardial I/R injury. These findings demonstrated that aspirin alleviated cardiac dysfunction by non-selective inhibition of COX enzymes and suppression of inflammatory cytokines such as IL-6 and TNF-α in the myocardium after I/R. This anti-inflammatory effect of aspirin was independent of its traditional anti-thrombotic effects.

### Aspirin aggravates gastric mucosal injury in response to myocardial ischemia/reperfusion by increasing inflammation

**FIGURE 2:**
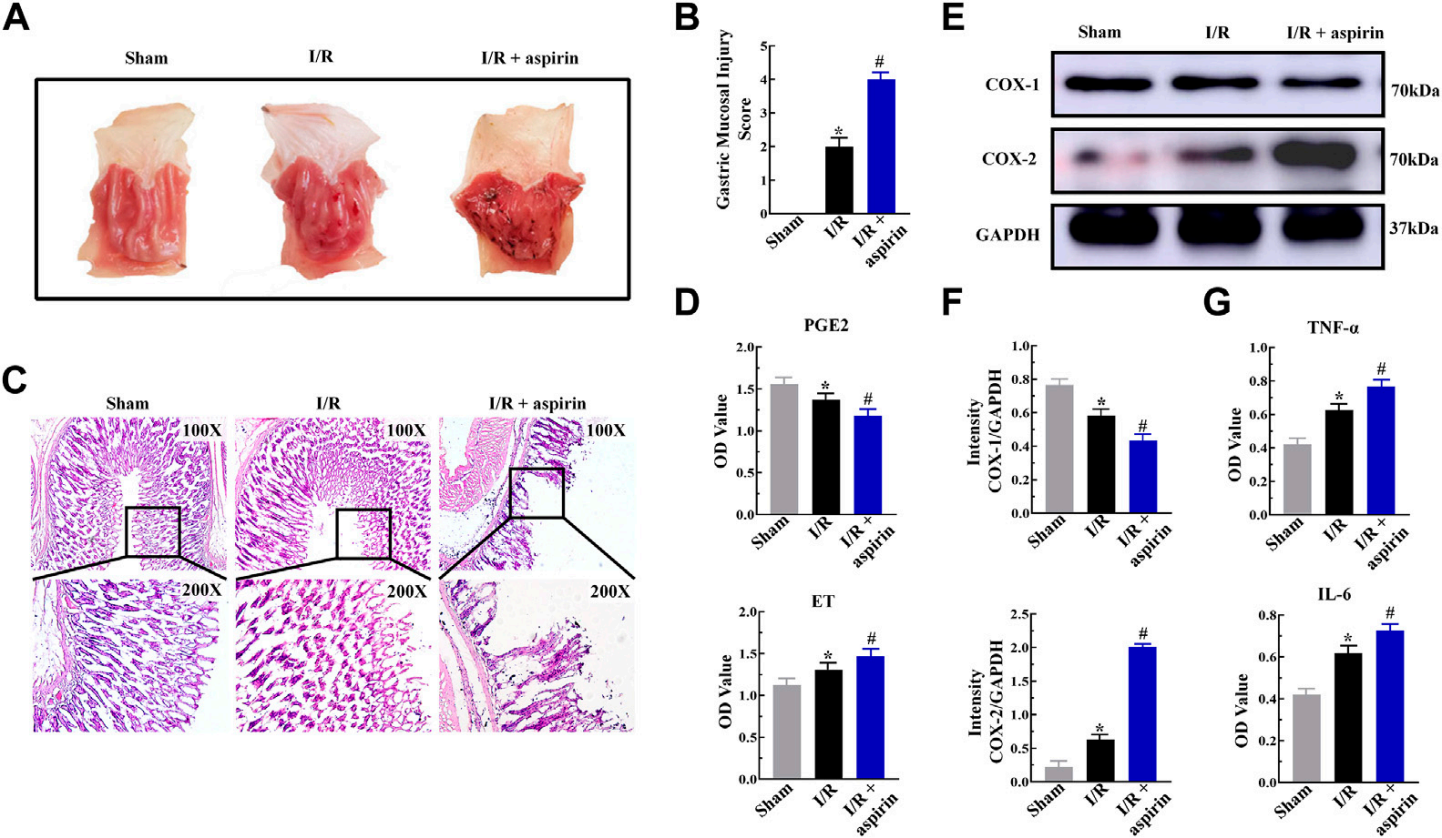
Aspirin aggravates gastric mucosal injury by inducing inflammation. **(A)** Representative images of the harvested stomach specimens from the sham, I/R, and I/R + 50 mg/kg aspirin group rats (*n* = 5 per group). **(B)** Byron gastric mucosal injury scores in the sham, I/R, and I/R + aspirin group rats (*n* = 5 per group). The scoring criteria are shown in Table 1. **(C)** Representative images (×100 and ×200) of the H&E-staining gastric mucosal tissue sections from the sham, I/R, and I/R + aspirin group rats (*n* = 5 per group). **(D)** ELISA assay results show the levels of prostaglandin E2 (PGE2) and endothelin (ET) in the gastric tissues of the sham, I/R, and I/R + aspirin group rats (*n* = 5). **(E,F)** Representative immunoblots and quantification analysis of COX-1 and COX-2 protein levels in the gastric tissues of the sham, I/R, and I/R + aspirin group rats (*n* = 5). **(G)** ELISA assay results show the levels of tumor necrosis factor-α (TNF-α) and interleukin-6 (IL-6) in the gastric tissues of the sham, I/R, and I/R + aspirin group rats (*n* = 5). The data are represented as means ± SD; * *p < 0.05* vs. the sham group; ^**#**^ *p < 0.05* vs. the I/R group.

Aspirin is a commonly prescribed drug for preventing secondary myocardial infraction with CHD. However, long-term use of aspirin causes gastric mucosal injury in CHD patients underwent PCI. In this study, myocardial I/R triggered gastric mucosal injury with significant glandular area hyperemia, mucosal edema and dot hemorrhage necrosis (Figure 2A). Furthermore, these lesions were further aggravated in the I/R + aspirin rats (Figure 2A). The histopathology data was confirmed by the gastric mucosal injury score results (*p < 0.05*) (Figure 2B). These results demonstrated that aspirin improved cardiac function after I/R injury but aggravated gastric mucosal injury. Furthermore, gastric tissue of I/R showed erosion of the surface epithelial cells, abscission and necrosis of gastric mucosa, rupture of the gastric glands, extensive edema and infiltration of the inflammatory cells in the mucosal layer, and all these pathological changes were aggravated in the I/R + aspirin rats (Figure 2C). Therefore, we analyzed the levels of PGE2 and endothelin (ET) which are involved in maintaining the gastric tissue integrity. Gastric mucosal tissues of I/R showed down-regulation of PGE2 and up-regulation of ET compared to the corresponding controls, while these changes were aggravated in aspirin treatment under I/R injury (*p < 0.05*) (Figure 2D). Based on this, we investigated the expression levels of inflammatory mediators. In comparison with Sham group, gastric mucosal tissues of I/R showed reduced expression of COX-1 and increased expression of COX-2, TNF-α and IL-6. All these changes were aggravated in I/R + aspirin rats (*p < 0.05*) (Figures 2E–G). Taken together, our results indicated that myocardial I/R resulted in both myocardial and gastric mucosal injury. Furthermore, treatment with aspirin significantly alleviated myocardial damage, while exacerbated gastric mucosal injury by enhancing inflammation.

### Gastrodin alleviates dual gastric mucosal injury induced by ischemia/reperfusion and aspirin *via* inhibiting inflammation and restoring prostaglandin E2 levels

**FIGURE 3:**
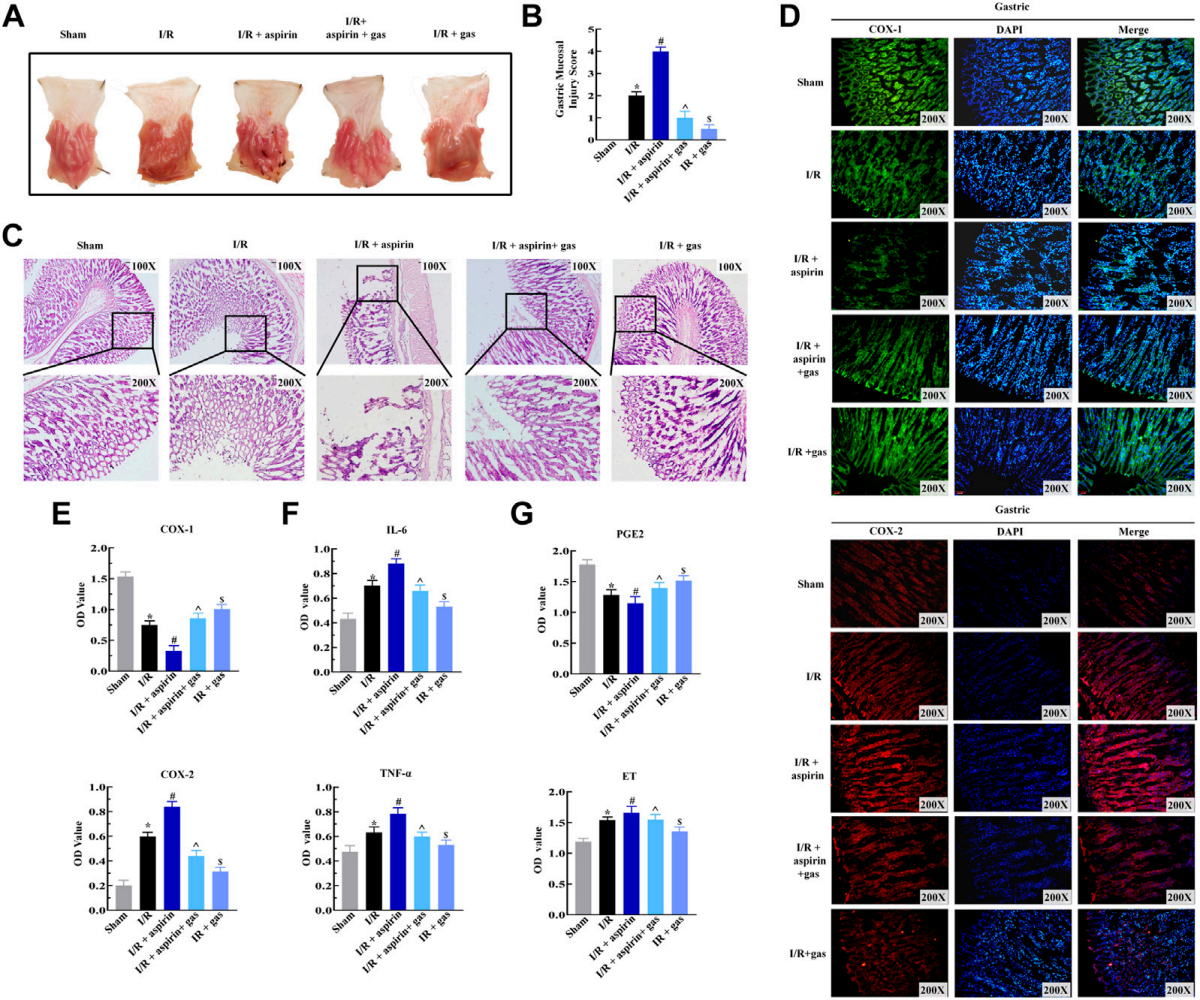
Gasrtodin alleviates dual gastric mucosal injury by suppressing inflammation. **(A)** Representative images of the harvest stomach specimens from the sham, I/R, I/R + aspirin, I/R + 50 mg/kg aspirin + 200 mg/kg gastrodin group, and I/R + 200 mg/kg gastrodin group rats (*n* = 5 per group). **(B)** Byron gastric mucosa injury scores of the sham, I/R, I/R + aspirin, I/R + aspirin + gastrodin group, and I/R + gastrodin group rats (*n* = 5 per group). The scoring criteria are shown in Table 1. **(C)** Representative images (×100 and ×200) of the H&E-staining gastric tissue sections from the sham, I/R, I/R + aspirin, I/R + aspirin + gastrodin group, and I/R + gastrodin group rats (*n* = 5 per group). **(D)** Representative immunofluorescence staining images (×200) show the COX-1 and COX-2 expression levels in the gastric tissues of the sham, I/R, I/R + aspirin, I/R + aspirin + gastrodin group, and I/R + gastrodin group rats (*n* = 5 per group). The green denotes COX-1, red denotes COX-2, and blue denotes the nucleus. **(E–G)** ELISA assay results show the levels of cyclooxygenase-1 (COX-1), cyclooxygenase-2 (COX-2), tumor necrosis factor-α (TNF-α), interleukin-6 (IL-6), prostaglandin E2 (PGE2) and endothelin (ET) in the gastric tissues of the sham, I/R, I/R + aspirin, I/R + aspirin + gastrodin group,and I/R + gastrodin group rats (*n* = 5 per group). The data are represented as means ± SD; * *p < 0.05* vs. the sham group; ^**#**^ *p < 0.05* vs. the I/R group; **^** *p < 0.05* vs. the I/R + aspirin group; ^&^ *p < 0.05* vs. the I/R + aspirin + gastrodin group.

Aspirin exerts both positive and negative effects on CHD. The anti-inflammatory effects of aspirin contributes to improve cardiac dysfunction, while its pro-inflammatory effects induce gastric mucosal injury. So we analyzed if combination of aspirin and gastrodin could alleviate gastric mucosal injury followed by myocardial I/R and aspirin treatment. In the absence of I/R, 50 mg/kg aspirin induced significant gastric mucosal injury in a time-dependent manner. Surprisingly, the gastric mucosal damage was evidently alleviated when 50, 200 and 400 mg/kg of gastrodin was used in combination with aspirin, respectively. Notably, the effects of gastrodin at 200 mg/kg was the most obvious (*p < 0.05*) (Supplementary Material S1). Therefore, we used 200 mg/kg gastrodin in combination with 50 mg/kg aspirin to explore its protective effect on gastric mucosa induced by myocardial I/R in the following study. We discovered that both individual gastrodin treatment and combined treatment with aspirin and gastrodin led to the reduced gastric mucosal lesions which were accompanied with mild hyperemia and sporadic lesions compared to individual aspirin treatment (Figure 3A). Byron gastric mucosal injury score results also showed the injury was significantly alleviated when applying both individual gastrodin treatment and combination treatment of aspirin and gastrodin compared to aspirin medication alone after myocardial I/R (*p < 0.05*) (Figure 3B). H&E staining of gastric mucosal tissues from combination of aspirin and gastrodin treatment demonstrated significantly reduction in the mucosal damage, edema and infiltration of inflammatory cells, as well as rupture of the gastric glands compared to aspirin medication alone (Figure 3C). Immunofluorescence and ELISA assay results demonstrated that COX-1 expression was reduced, while expression levels of COX-2, TNF-α and IL-6 were significantly elevated in the gastric tissues of aspirin medication alone. However, these effects were reversed to nearly normal levels in the gastric tissues of combination of aspirin and gastrodin treatment (*p < 0.05*) (Figures 3D–F). ELISA assay showed that the dysregulation of PGE2 and ET expression in the gastric tissues of the individual aspirin treatment was restored back to normal after combination of aspirin and gastrodin treatment in myocardial I/R (*p < 0.05*) (Figure 3G). In conclusion, combined treatment with aspirin and gastrodin after myocardial I/R effectively alleviated gastric mucosal injury caused by aspirin administration alone through inhibition of inflammation and restoration of PGE2 levels.

### Combined treatment with aspirin and gastrodin mitigates cardiac dysfunction induced by myocardial ischemia/reperfusion by suppressing inflammation and increasing cyclooxygenase-1 expression

**FIGURE 4:**
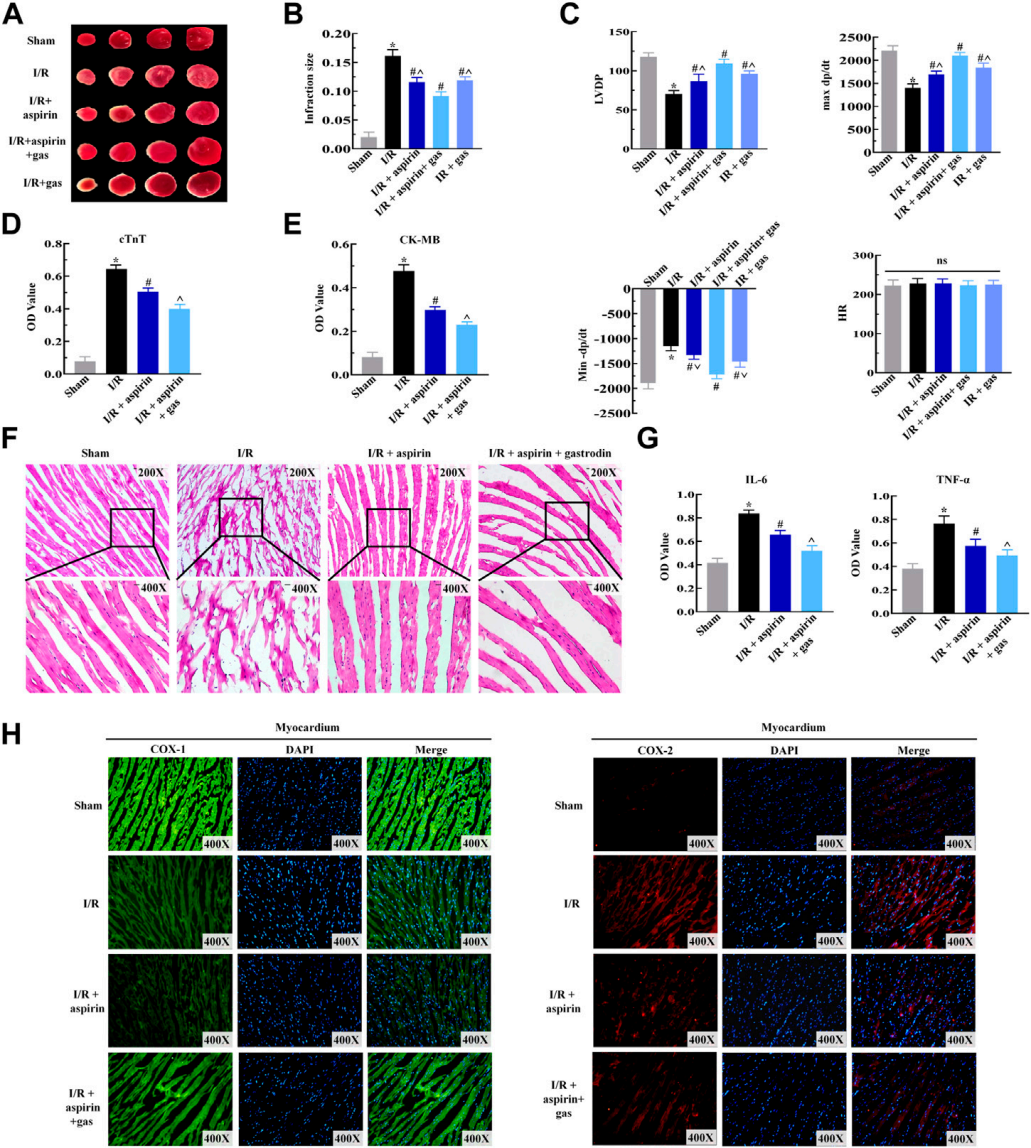
Combined therapy with aspirin and gastrodin alleviates cardiac dysfunction induced by myocardial I/R. **(A,B)** Representative TTC staining images of the myocardium from the sham, I/R, I/R + aspirin, I/R + aspirin + gastrodin group, and I/R + gastrodin group rats (*n* = 5 per group). Quantification of the infarct size based on TTC staining of the myocardium from the sham, I/R, I/R + aspirin, I/R + aspirin + gastrodin group, and I/R + gastrodin group rats (*n* = 5 per group) is also shown. **(C)** Laggendorff *in vitro* cardiac perfusion measurements show the left ventricular developed pressure (LVDP), maximum and minimum rates of pressure development (dP/dt max and dP/dt min) and heart rate (HR) in the sham, I/R, I/R + aspirin, I/R + aspirin + gastrodin group, and I/R + gastrodin group rats (*n* = 5 per group). **(D,E)** ELISA assay results show the serum creatine kinase-MB (CK-MB) and cardiac troponin T (cTnT) levels in the sham, I/R, I/R + aspirin, and I/R + aspirin + gastrodin group rats (*n* = 5 per group). **(F)** Representative H&E-staining images (×200 and ×400) show the myocardial histology and morphological characteristics in the sham, I/R, I/R + aspirin, and I/R + aspirin + gastrodin group rats (*n* = 5 per group). **(G)** ELISA assay results show the tumor necrosis factor-α (TNF-α) and interleukin-6 (IL-6) levels in the myocardial tissues of the sham, I/R, I/R + aspirin, and I/R + aspirin + gastrodin group rats (*n* = 5 per group). The samples for ELISA were harvested 2 h post-reperfusion. **(H)** Representative immunofluorescence staining images (×400) show the COX-1 and COX-2 expression levels in the heart tissue of the sham, I/R, I/R + aspirin, and I/R + aspirin + gastrodin group rats (*n* = 5 per group). Green represents COX-1; red represents COX-2; blue represents the nucleus. The data are represented as means ± SD; * *p < 0.05* vs. the sham group, ^**#**^ *p < 0.05* vs. the I/R group, **^** *p < 0.05* vs. the I/R + aspirin + gastrodin group.

Previously, we confirmed the protective effect of aspirin combined with gastrodin on gastric mucosal injury. To further estimate the role of combination drugs on cardiac dysfunctions induced by I/R, rats were pretreated with aspirin and gastrodin for 3 days prior to I/R injury. After establishing I/R model, we were dramatically surprised that combination of aspirin and gastrodin further led to effectively reduce infarct size compared with aspirin administration alone (*p < 0.05*) (Figures 4A,B). On the basis of these results, we examined the effect of combination medication on cardiac function. The results indicated that aspirin combined with gastrodin could more forcefully recover the level of LVDP and ±dP/dt when compared with aspirin alone (*p < 0.05*) (Figure 4C). Based on the determination of myocardial enzyme content in the serum, combination drugs significantly reduced CK-MB and cTnT release from infarcted myocardium (*p < 0.05*) (Figures 4D,E). Further observation under the microscope revealed combination of aspirin and gastrodin could recover the disorder of myocardial structure, neutrophils infiltration, interstitial edema, cardiomyocyte necrosis and nuclear dissolution, of which the effect was better than aspirin (Figure 4F). Subsequently, we assessed the expressions of COX and inflammatory factors in heart tissues to further clarify the underlying mechanism by which combined drugs exert effects on cardiac function injury. The results demonstrated that aspirin combined with gastrodin could further down-regulate the expression of COX-2, IL-6 and TNF-α while restoring the variation of COX-1 (*p < 0.05*) (Figures 4G,H). These findings indicated that combination of aspirin and gastrodin dramatically improved myocardial I/R-induced cardiac dysfunctions more than aspirin administration alone, which was achieved by restraining inflammation in the myocardium.

### Combined treatment with aspirin and gastrodin suppresses pyroptosis in both the myocardium and the stomach

**FIGURE 5:**
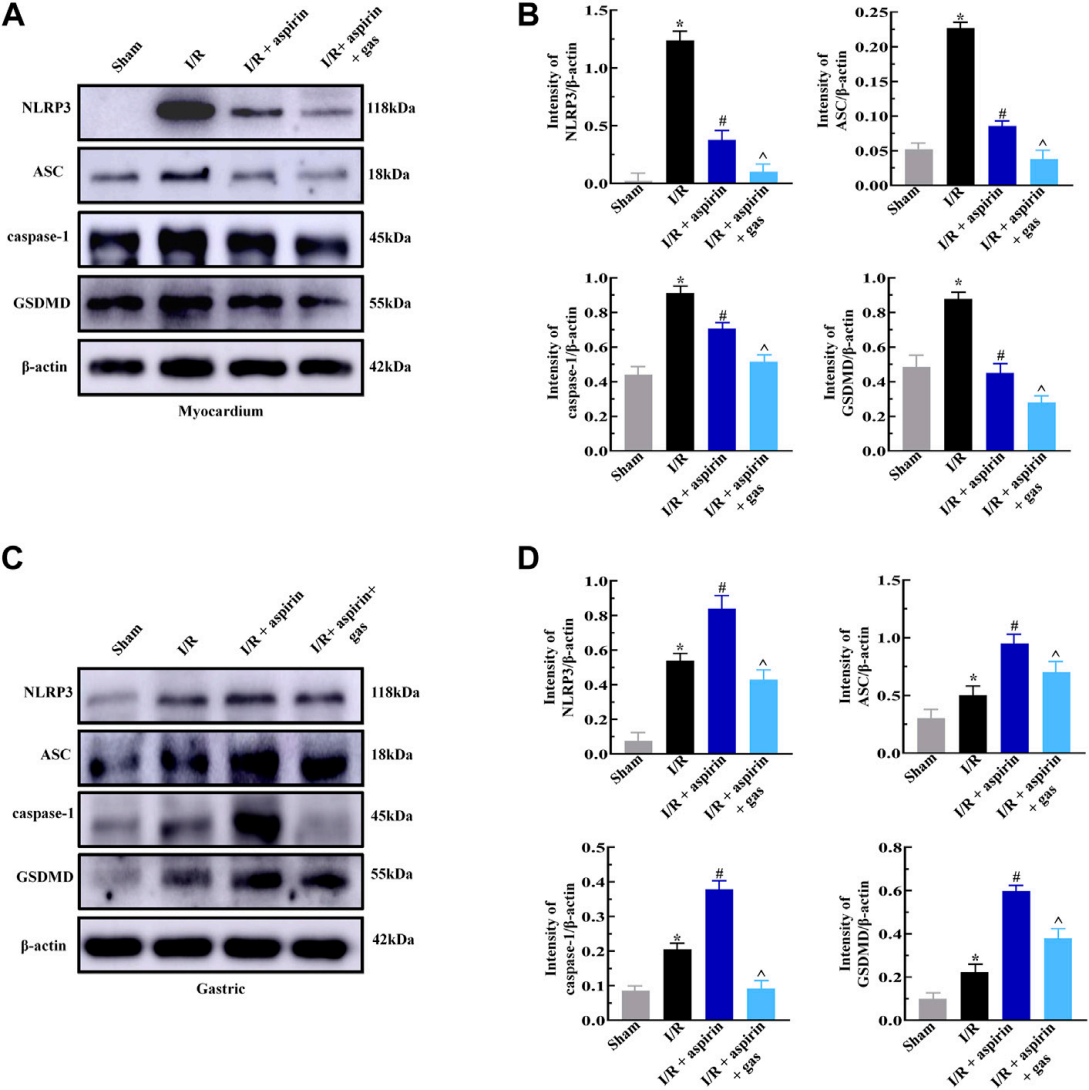
Combined treatment with aspirin and gastrodin protects against cardiac and gastric mucosal injury by suppressing pyroptosis. **(A,B)** Representative immunoblots and quantification analysis of the NLRP3, ASC, caspase-1, and GSDMD protein levels in the myocardium of the sham, I/R, I/R + aspirin, and I/R + aspirin + gastrodin group rats (*n* = 5 per group). **(C,D)** Representative immunoblots and the quantification analysis of the NLRP3, ASC, caspase-1, and GSDMD protein expression levels in the gastric tissues of the sham, I/R, I/R + aspirin, and I/R + aspirin + gastrodin group rats (*n* = 5 per group). The data are represented as means ± SD; * *p < 0.05* vs. the sham group; ^**#**^ *p < 0.05* vs. the I/R group; **^** *p < 0.05* vs. the I/R + aspirin group.

Next, we explored whether combined treatment with aspirin and gastrodin could protect against pyroptosis in the myocardium and gastric tissues by analyzing the expression levels of pyroptosis-related proteins, namely, NLRP3, ASC, caspase-1 and GSDMD. The expression levels of NLRP3, ASC, caspase-1 and GSDMD were significantly increased after I/R, while those proteins were significantly reduced in myocardium of both combination of aspirin and gastrodin treatment and individual aspirin treatment (*p < 0.05*) (Figures 5A,B). Furthermore, the levels of NLRP3, ASC, caspase-1 and GSDMD were significantly decreased in the gastric tissues of combination of aspirin and gastrodin treatment than those with individual aspirin treatment (*p < 0.05*) (Figures 5C,D). These results demonstrated that aspirin inhibited pyroptosis in the myocardium while promoting pyroptosis in the gastric tissues after myocardial I/R. However, combined treatment with aspirin and gastrodin inhibited against myocardial I/R-induced pyroptosis in both the myocardium and the gastric tissues.

### Combined therapy with aspirin and gastrodin does not affect anti-thrombotic effects of aspirin

**FIGURE 6:**
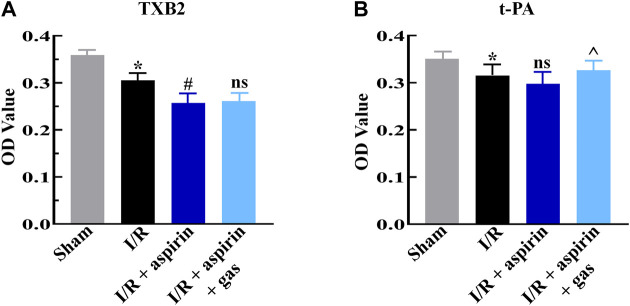
Combined treatment with aspirin and gastrodin shows effective antithrombotic effects. **(A,B)** ELISA assay results show the serum levels of thromboxane B2 (TXB2) and tissue-type plasminogen activator (t-PA) in the sham, I/R, I/R + aspirin, and I/R + aspirin + gastrodin group rats (*n* = 5 per group). The samples were obtained 2 h post-reperfusion. The data are represented as means ± SD; * *p < 0.05* vs. the sham group; ^**#**^ *p < 0.05* vs. the I/R group; **^** *p < 0.05* vs. the I/R + aspirin group.

Furthermore, we discussed the effects of combined treatment with aspirin and gastrodin on anti-thrombotic function of aspirin by estimating the serum levels of thromboxane B2 (TXB2) and tissue-type plasminogen activator (t-PA) levels. The serum TXB2 level was significantly down-regulated (*p < 0.05*), while t-PA level was not altered in the individual aspirin treatment compared to without aspirin treatment in myocardial I/R (*p > 0.05*) (Figures 6A,B). However, the expression level of TXB2 in combination of aspirin and gastrodin treatment was similar to those in the individual aspirin treatment (*p > 0.05*) (Figures 6A,B); meanwhile, increased t-PA expression level was revealed in combination of aspirin and gastrodin treatment compared with the individual aspirin treatment (*p < 0.05*) (Figures 6A,B). This demonstrated that gastrodin did not inhibit the anti-thrombotic effects of aspirin.

## Discussion

CHD patients are clinically treated with drugs such as aspirin, clopidogrel (Redfors et al., 2022), low molecular weight heparin or nitroglycerin (El-Sabrout et al., 2022). Aspirin is frequently prescribed in the perioperative period of PCI among these drugs. Patients with AMI often receive anti-thrombotic therapy with aspirin at doses of 250–500 mg/kg before PCI (Calvieri et al., 2022). Furthermore, long term use of aspirin is used to prevent secondary myocardial infarction after PCI. However, long term use of aspirin is associated with adverse effects such as gastric mucosal bleeding and injury as well as anaphylactic reactions (Wallace, 1997; Wang et al., 2021). We also demonstrated that aspirin caused gastric mucosal injury within 1–5 days, along with hyperemia and edema, rupture of the gastric glands, extensive edema and infiltration of inflammatory cells.

Aspirin is a classical non-steroidal anti-inflammatory drug that does not interact with any surface or intracellular receptors, but exerts its activity through non-specific irreversible inhibition of COX-1 and COX-2 via acetylation. It is well established that COX-2 expression, induced by inflammatory cytokines, hormones and growth factors, plays a significant role in acute stress, inflammation and infection. COX-1 regulates gastric mucosal blood flow and synthesis of prostaglandins and TXA2. The inhibition of COX-2 activity through acetylation by aspirin reduces biosynthesis of prostaglandins and inflammatory responses. Irreversible inhibition of COX-1 by aspirin through acetylation of serine 530 down-regulates TXA2 and PGE2 expression levels, thereby increasing gastric mucosal injury and reducing platelet activity (Zareef et al., 2022). However, mechanisms by which aspirin induces gastric mucosal injury is not clear. Wallace et al. suggested that aspirin treatment caused gastric injury through simultaneously inhibiting COX-1 and COX-2 (Wallace et al., 2000). However, other studies suggested that aspirin induced gastric mucosal injury by inhibiting COX-1 and activating COX-2 (Davies et al., 1997). Our results demonstrated that aspirin down-regulated the expression of COX-1 and COX-1-dependent PGE2 in the gastric tissues. However, aspirin significantly up-regulated COX-2 expression in the stomach accompanied by elevated expression of inflammatory cytokines (TNF-α and IL-6). This indicated that aspirin promoted inflammation in the gastric mucosa. Furthermore, aspirin induced vasoconstriction in the gastric mucosa by increasing the levels of ET, thereby reducing blood supply to the gastric mucosa. Therefore, our study demonstrated that aspirin caused gastric mucosal injury by reducing PGE2, an essential factor to maintain mucosal barrier integrity, and upregulating inflammation.

Although long term use of aspirin causes significant gastric mucosal injury, it is still the preferred drug for treating CHD because it is highly effective in suppressing thrombosis through suppression of COX-1 and TXA2 (Basili et al., 2014). A recent randomized controlled trial demonstrated that aspirin protected the myocardium from I/R injury by inhibiting cardiomyocytes apoptosis (Wu et al., 2021). This suggested that aspirin protects against myocardial dysfunction through multiple mechanisms, such as suppression of thrombosis and cardiomyocyte apoptosis. In this study, treatment with 50 mg/kg aspirin led to the reduced the myocardial infraction size and serum levels of myocardial enzymes, namely, CK-MB and cTnT, which indicated aspirin-induced protection of cardiomyocytes from death in response to I/R. Histological analysis also showed that aspirin significantly suppressed the cardiomyocyts death, edema and inflammatory cell infiltration. Furthermore, we revealed the effects of aspirin on *ex vivo* cardiac function by evaluating LVDP, ±dP/dt and HR. Aspirin significantly normalized LVDP and ±dP/dt in response to I/R. So, what are the underlying mechanisms by which aspirin exerts a protective role in cardiac function? In addition to the anti-platelet function, aspirin exerts anti-inflammatory effects as its one of the important pharmacological actions. Therefore, we analyzed the role of aspirin in myocardial inflammation which plays a critical role in myocardial I/R injury. We confirmed that aspirin significantly reduced the expression of COX-1, COX-2, TNF-α and IL-6 in myocardial tissues, which indicates that aspirin inhibited inflammation in addition to the anti-thrombotic effects.

Aspirin protects myocardium from I/R injury but causes gastric mucosal injury. This demonstrates that the effects of aspirin are selective on different organs. Our data showed that I/R induced myocardial inflammation by producting COX-2, TNF-α and IL-6. However, aspirin inhibited inflammatory production, thereby protecting the cardiac function in response to I/R. Furthermore, aspirin reduced TXA2 levels, which sequentially inhibited thrombosis. Therefore, aspirin played a positive role in the heart during I/R. However, the acidic properties of aspirin promoted gastric mucosal damage. Aspirin is inclined to entry into the surface epithelial cells in a highly acidic gastric environment via its non-ionized lipophilic form, which sequentially induces mucosal injury. Furthermore, aspirin decreases PGE2 and TXA2 expression as well as the synthesis and secretion of mucus and bicarbonate, which contributes to the increased risk of gastric mucosal bleeding. Our results were consistent with previously published findings of gastric mucosal injury. Aspirin also induced the expression levels of COX-2, TNF-α and IL-6, and promoted inflammation-induced gastric mucosal injury. Taken together, these data suggested that the diverse effects of aspirin on the heart and stomach in response to I/R, which may be associated with its regulation of the inflammatory response, coagulation and prostaglandin expression.

ECA is usually prescribed to prevent the negative effects of aspirin on the gastric mucosa. In order to effectively protect gastric mucosa, ECA contains cellulose or silicon that prevents disintegration of the tablet in the stomach. However, a recent systematic review reported that ECA treatment was not effective in protecting the gastric mucosa. Therefore, therapeutic regimen that aspirin treatment should be used in combination with gastric protective agents was put forward, including combination with proton pump inhibitors such as omeprazole, rabeprazole and others (Fitzgerald and Corley, 2019). However, the proton pump inhibitors are associated with adverse effects such as hypomagnesemia and osteoporosis (Bridoux et al., 2022). One of the options to overcome these issues is to combine aspirin with natural medicines that may avoid the adverse effects. Gastrodin has been widely used in Traditional Chinese Medicine. Researchers have reported that gastrodin exerts therapeutic effects on a variety of diseases, including hypertension, angina pectoris, cardiac hypertrophy, vertigo, epilepsy, etc (Li et al., 2019). Notably, gastrodin ameliorates the death of cardiomyocytes by triggering autophagy (Fu et al., 2018), suppressing inflammation and apoptosis (Han et al., 2019), and inhibiting pyroptosis (Sun et al., 2019). Thus, gastrodin may play an important role in treatment of cardiovascular diseases. Furthermore, few adverse reactions of gastrodin have been reported and gastrodin takes effects in a wide range of concentrations. Recently, it is reported that the combination of gastrodin and betahistine improves betahistine-induced gastrointestinal dysfunction *via* regulating the secretion of gastric acid (Zhang et al., 2022b). Zheng et al. believed that gastric mucosal injury induced by borneol could be mitigated when gastrodin was combined with borneol (Cai et al., 2014). Therefore, we evaluated the combination of aspirin and gastrodin as a novel therapeutic regimen in myocardial I/R injury. The combination of aspirin and gastrodin significantly reduced the adverse effects of aspirin including gastric bleeding and edema. We tested different doses of gastrodin (50, 200 and 400 mg/kg) in combination with aspirin and found that 200 mg/kg dose was the most optimal. Next, the study compared the efficacy of the combinatorial drug regimen with that of aspirin in myocardial I/R injury and evaluated its effects on the gastric mucosal injury induced by aspirin. The combined treatment with aspirin and gastrodin significantly alleviated gastric mucosal damage. Histologically, the combination drug regimen protected gastric mucosal integrity by normalizing the expression of PGE2 and COX-1, which was adversely affected by aspirin alone. The combinatorial regimen of aspirin and gastrodin also suppressed expression of ET, as well as decreased inflammation by inhibition the expression of COX-2, TNF-α and IL-6 in the gastric tissues. Therefore, the combination of aspirin and gastrodin could alleviate gastric mucosal injury, which is consistent with the previous view that gastrodin has a protective effect on gastrointestinal tract.

This study demonstrated that aspirin mitigated myocardial infraction size and improved cardiac function. In a previous study, we reported that gastrodin alleviated myocardial I/R injury by inhibiting autophagy (Tao et al., 2021). Here, we observed that the combined treatment synergistically protected the myocardial structure and function by preventing myocardial cell edema, rupture and necrosis after I/R. Functionally, the combined drug regimen normalized the levels of LVDP and ±dP/dt, thereby improving myocardial contractility by suppressing the levels of COX-2, TNF-α and IL-6 in the myocardial tissues. Therefore, our results demonstrated that the combination treatment with aspirin and gastrodin was effective in protecting the heart from I/R injury.

TXA2 plays a vital role in thrombosis and is responsible for the occlusion of blood vessels in CHD. Therefore, pharmacological inhibition of TXA2 is an important target for CHD and related ischemic heart diseases (Patrono and Baigent, 2019). Aspirin is an important inhibitor of TXA2. Here, we demonstrated that gastrodin does not affect the anti-thrombotic functions of aspirin. The levels of TXB2, a stable metabolite of TXA2 (Kou et al., 2018), were similar in rats treated with aspirin alone and the combined drug regimen of aspirin and gastrodin. t-PA is a serine protease and a physiological agonist of the fibrinolytic system. It acts by converting plasminogen to plasmin and helps dissolve or prevent blood clots (Loscalzo and Braunwald, 1988). Our data suggested that the combination of aspirin and gastrodin increased t-PA levels. This demonstrated that gastrodin may act in synergy with aspirin to protect against thrombosis.

In this study, we demonstrated that the combination of aspirin and gastrodin effectively suppressed inflammation to protect heart and stomach against I/R-induced injury. Pyroptosis refers to programmed cell death triggered by the inflammasomes and executed by gasdermin proteins. The main characteristics of pyroptosis include cell swelling, membrane perforation and the release of cellular contents (Rao et al., 2022). The classical pyroptosis pathway is mediated by the NLRP3/caspase-1/GSDMD pathway. Pyroptosis is induced through cleavage of GSDMD by activated caspase-1 (Qian et al., 2021; Chen et al., 2022; Zhang et al., 2022a). We confirmed that aspirin protected myocardial tissue in response to I/R by decreasing pyroptosis-related proteins, including NLRP3, ASC and GSDMD. However, the expression levels of these pyroptosis-related proteins were significantly high in aspirin-treated stomach tissues and likely contributed to the extensive gastric mucosal injury. On the other hand, the levels of pyroptosis-related proteins were significantly low in both heart and stomach tissues treated with both aspirin and gastrodin. This demonstrated that combined treatment of aspirin and gastrodin suppressed pyroptosis in stomach and heart tissues in response to I/R.

Our study demonstrated that myocardial I/R induced gastric mucosal injury. Furthermore, myocardial I/R down-regulated PGE2 expression and up-regulated ET expression in the gastric mucosa, thereby affecting the integrity of the gastric mucosal protective barrier and optimal blood flow. Moreover, I/R induced inflammation with increased expression levels of COX-2, TNF-α and IL-6 in the gastric mucosa. Therefore, we postulate that TNF-α and IL-6 produced by the damaged myocardial tissues in response to I/R reach the stomach through circulation and induce inflammation-related damage to the gastric mucosa. Furthermore, myocardial I/R injury impedes heart pumping function, thereby further exacerbating hypoxic-ischemic gastric injury.

## Conclusion

**FIGURE 7:**
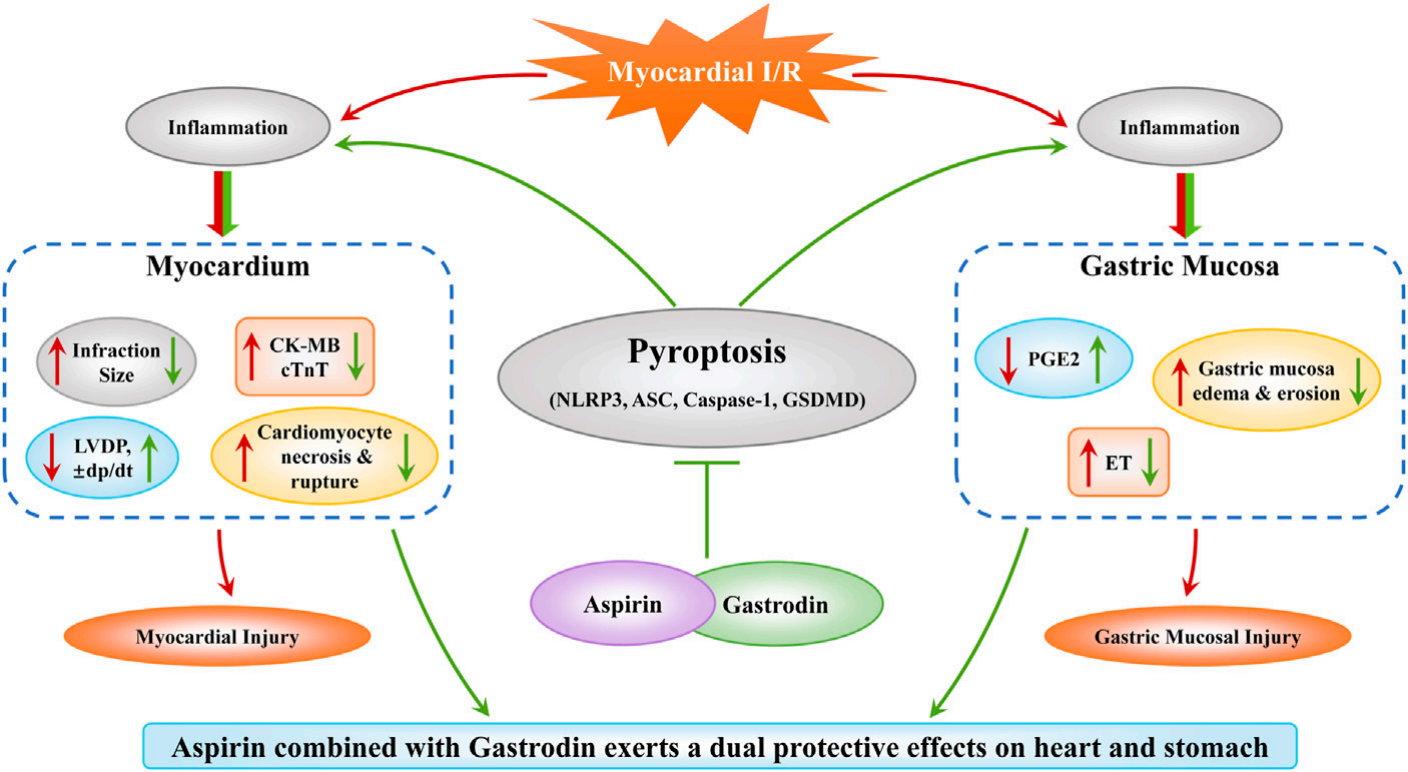
Combined treatment with aspirin and gastrodin protects against cardiac and gastric mucosal injury in response to myocardial ischemia/reperfusion. Myocardial I/R causes both myocardial and gastric mucosal damage. However, combined treatment with aspirin and gastrodin exerts a dual protective effect on the myocardium and gastric mucosa by inhibiting pyroptosis. In the heart, the combined treatment of aspirin and gastrodin reduced the myocardial infarct size and myocardial cell apoptosis, down-regulated the release of CK-MB and cTnT into the blood, and restored cardiac function indicators such as LVDP, ±dp/dt. In the stomach, the combined treatment of aspirin and gastrodin inhibited the inflammatory response, reduced the gastric mucosal edema, necrosis and exfoliation, and restored the near normal expression levels of PGE2 and ET.

In conclusion, our study demonstrated that the combined treatment with aspirin and gastrodin protected both heart and gastric mucosal tissues after myocardial I/R by suppressing inflammation and pyroptosis, while preserving the anti-thrombotic functions of aspirin (Figure 7). Our study postulates a new therapeutic strategy for alleviating myocardial and gastric mucosal injury in CHD through combined therapy with aspirin and gastrodin. Next, further mechanistic exploration needs to be carried out.

## Data Availability

The original contributions presented in the study are included in the article/Supplementary Material, further inquiries can be directed to the corresponding authors.
