# Supplementary material for: Aspirin in combination with gastrodin protects cardiac function and mitigates gastric mucosal injury in response to myocardial ischemia/reperfusion
Source: Front Pharmacol. 2022 Sep 27;13:995102. doi: 10.3389/fphar.2022.995102 (PMC9553090; doi:10.3389/fphar.2022.995102)
Supplement: Supplementary file 1 [file DataSheet1.docx]

Supplementary Material

## Supplementary Figures

**
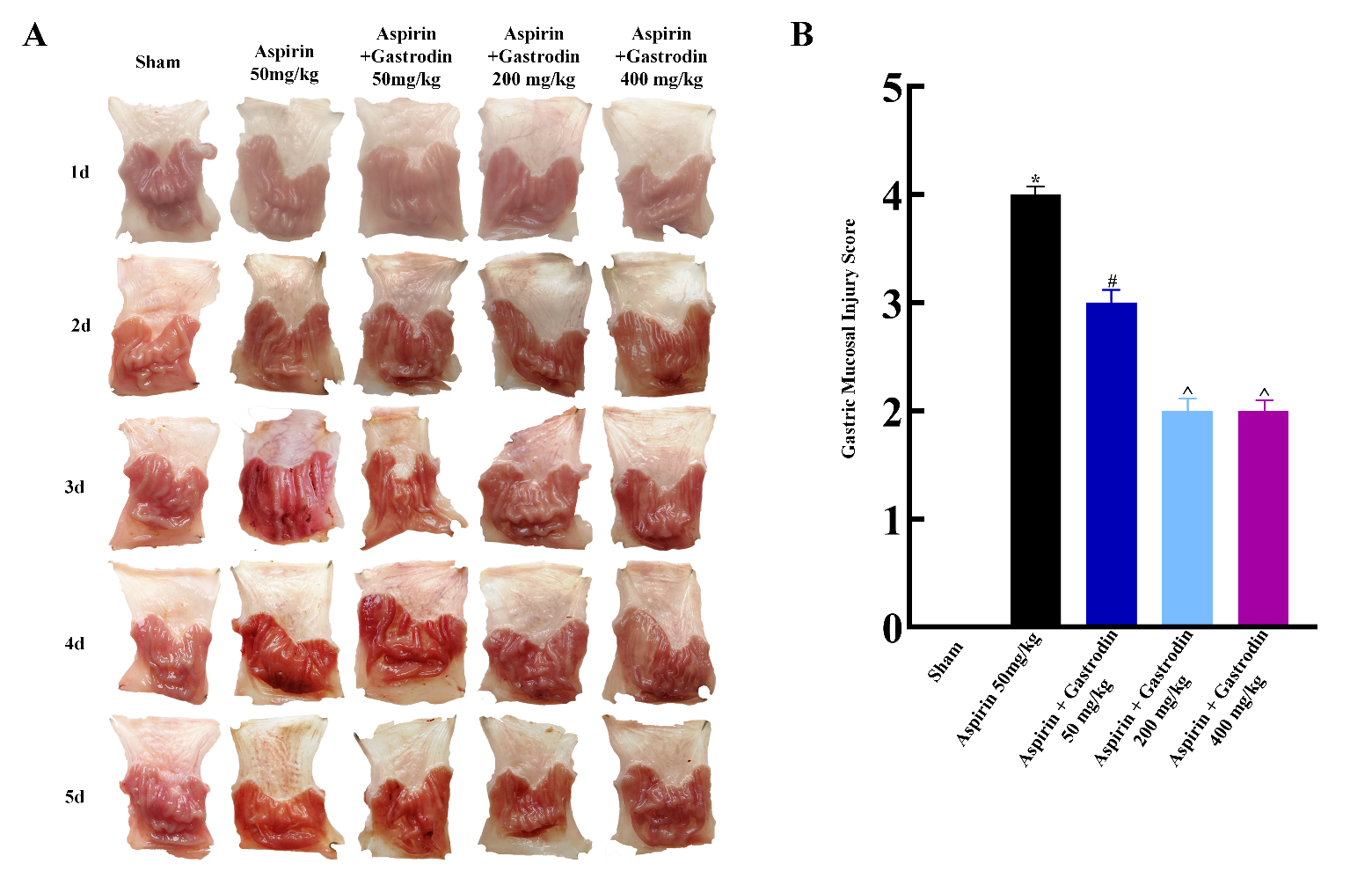
**

**SUPPLEMENTARY FIGURE 1.**

**FIGURE S1** Protective effect of Aspirin combined with Gastrodin on gastric mucosal injury. **(A)** Images of stomach specimens from mice of each group. Sham group: 0.9%NaCl treatment; Aspirin alone group: mice were treated with 50 mg/kg Aspirin by gavage; Aspirin + Gastrodin group: in addition to Aspirin, mice were also treated with 50, 200, 400 mg/kg Gastrodin (n=5). **(B)** Byron gastric mucosa injury score was used to evaluate gastric mucosa damage. The scoring criteria are shown in Table 1. Data are presented as means ± SD. ******p < 0.05* vs. the sham group, **^#^***p < 0.05* vs. the Aspirin group, **^***p < 0.05* vs. Aspirin + Gastrodin 50 mg/kg group.

**SUPPLEMENTARY RESULTS**

In order to explore the protective effect of Aspirin combined with Gastrodin on Aspirin-induced gastric mucosal injury, different concentrations of Gastrodin 50mg/kg, 200mg/kg and 400mg/kg were used in combination with 50mg/kg Aspirin to treatment gastric mucosal injury. After taking Aspirin for 1-5 days, different degrees of damage to the gastric mucosa appeared, which showed obvious time dependence, manifested as gastric mucosal congestion, hemorrhage, and edema. After taking Aspirin for 1-2 days, the gastric mucosa showed only mild congestion and edema. However, on the third day of taking Aspirin, obvious edema, hyperemia, and bleeding spots were seen on the gastric mucosa. At 4-5 days, the gastric mucosal injury was the most serious, showing a large area of hyperemia and edema with bleeding spots. Subsequently, we combined 50mg/kg, 200mg/kg, 400mg/kg of Gastrodin and Aspirin, and discovered that different concentrations of Gastrodin combined with Aspirin could significantly alleviate gastric mucosal congestion, hemorrhage and edema, indicating that combination therapy protects gastric mucosa from Aspirin-induced damage (**Figure S1A**). However, from the three effective doses of Gastrodin, 200 mg/kg and 400 mg/kg combined with Aspirin had better protective effect on gastric mucosal injury, which restores gastric mucosal congestion, hemorrhage, and edema to near-normal levels, while 50 mg/kg had a poor protective effect. The Byron gastric mucosal injury score also showed the same results (**Figure S1B**).
